# The Inside Scoop: What We Learnt About Getting into Academic Publishing During Our Editorial Internship

**DOI:** 10.1007/s40670-023-01961-2

**Published:** 2023-12-20

**Authors:** Church H. R, Govender L

**Affiliations:** 1 Medical Education, University of Nottingham, Nottingham, UK; 2 Division of Anatomical Pathology, Faculty of Health Sciences, University of Cape Town, Cape Town, South Africa; 3 Faculty of Medicine and Health Sciences, Medical School, University of Nottingham, Queen’s Medical Centre, Room B87, Nottingham, NG7 2UH UK

**Keywords:** Academic Writing, Authorship, Editing, Manuscript, Peer-review, Academic Journal, Publication

## Abstract

The world of publication can seem intimidating and closed to the newcomer. How then does one even begin to get a foot in the door? In this paper, the authors draw from the literature and their recent lived experience as editorial interns to consider this challenge under the theme of access, and how it overlaps with the various components of academic publication. The main three components of the publication ‘machine’ are discussed in this article, *authoring*, *reviewing*, and *editing*. These are preceded by the first, and arguably foundational, interaction with academic journal publishing—reading. Without reading articles across different journals, and even in different disciplines, understanding the breadth of scholarship and its purpose is impossible. The subsequent components of *authoring*, *reviewing*, and *editing*, which are all enhanced by ongoing familiarity with current literature through further reading, are considered in further detail in the remainder of this article, with practical advice provided as to how to gain access and experience in each of these areas, for example, writing non-research article manuscripts, engaging in collaborative peer review, and applying for editorial opportunities (with perseverance) when the opportunity presents itself. Medical education publication can seem daunting and closed to entry-level academics. This article is written to dispel this view, and challenges the notion that the world of publication is reserved for experts only. On the contrary, newcomers to the field are essential for academic publications to retain relevance, dynamism, and innovation particularly in the face of the changing landscape of medical education.

## Introduction

As with many disciplines, medical education publication opportunities are often perceived as being reserved for the already established and well-connected [1]. Newcomers to this area of academia find themselves in the self-fulfilling prophecy of being unable to secure the currency of ‘experience’ which is the key to accessing more formal roles in the academic publication system. As two early-careers educators carving our own pathway into the world of publication, we recognise the many opportunities that are available, although this was not always the case. We both felt the need to learn more about the dynamics of publication, and as such applied for an editorial internship with a reputable academic journal. Through the internship, we have been given the opportunity to become more familiar with the academic publication process and recognise that there are ways in which early-career educationalists can develop skills and establish themselves within academic publication, but these are not well advertised to interested parties.

We recognise that our intern experience allowed us access to a world which might seem steeped in hierarchy; those at the peaks of their careers most likely to be welcomed and are certainly afforded the most influential roles. As two early-career academics, we acknowledge the great privilege of access that we have enjoyed, and in turn feel responsibility to share our findings with the wider community [2]. We offer this article to disseminate our insights, encourage, and highlight opportunities or others at similar stages of their academic careers with the hope of encouraging and inviting newcomers to the publication community. While PhD-trained medical educators may have experience with publishing as part of their graduate school or postdoctoral training, clinically trained faculty may vary widely in whether or not they have publishing experience. Acknowledging this diversity of publication experience, we formulate this manuscript for all those keen to engage with the world of academic publication, and encourage those interested to seek out both experience and training to learn *how* to undertake peer review in order to protect the rigour and standards associated with this method of publication. For these scholars, two things are essential to one’s understanding of publication: ‘what can I do?’ and ‘how do I make that happen’?

## Conceptual Overview of Academic Peer-Reviewed Publication

**Fig. 1:**
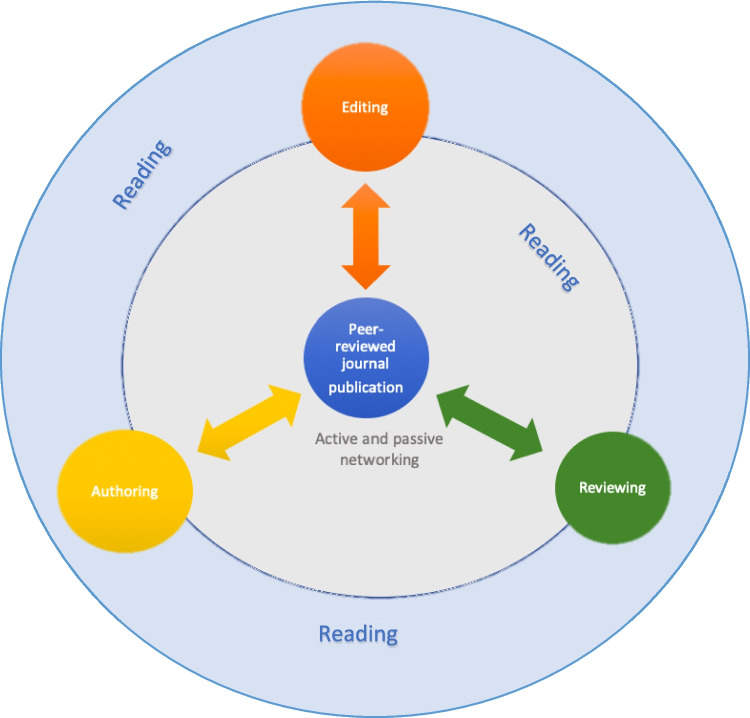
Roles in academic publication

Figure 1 offers a conceptual overview of academic peer-reviewed publication—the three main ‘cogs’ of which are authoring, editing, and reviewing: all of which is informed and influenced by reading and networking. From the lens of a single publication, the cogs are aligned and drive the next stage of the paper to fruition, but each step involves different operators—writers, editors, and reviewers. It is not always a linear process; the gears might shift, and the process reverses whilst a manuscript is passed iteratively between different stages of editing and reviewing, or reviewing and authoring during revision.

Drawing parallels with the journey of a single manuscript through the publication machine, so too is the experiential journey of a person working, and ascending the ladder of seniority, within publication. The experiences of reader, author, and reviewer all feed into the subsequent role—authors reflect on good and poor practice with article composition, and reviewers consider how they might have valued constructive feedback on their own submissions and how this can be phrased kindly, but fairly to those whose work they are now reviewing. Editors accumulate all this experience—weighing the perspectives of different reviewers, crafting a careful response to the author submitting the manuscript, and considering whether the readership of the journal might value this article if accepted.

### A Note on Reading

Stephen King, a highly successful and prolific writer, in his book *On Writing: A Memoir of the Craft*, offers sound advice for would-be writers. When asked to give an abbreviated version of his book to audiences, his statement is simple: ‘Write a lot and read a lot.’ [3]. Although academic publication may seem a long way from the fiction novels that Stephen King is known for, the advice still holds true. If one is to successfully author papers, reading and writing are both required, and tend to inform each other. For those embarking on their academic writing journey, it is important to understand what the end destination ‘looks like’. Reading widely and consistently allows one to gain familiarity with the high-impact journals in the field, and the kind of manuscripts which make it through to publication [4].

Reading published articles of the same format and from the same journal is invaluable regarding assessing whether your content is appropriate and relevant. For those who may be unclear where to begin their reading, consider a list of important journals in the field [5] or consult with peers about which journals or articles they think may be of interest to you. Admittedly, the need for institutional access or high membership fees might prevent newcomers to the field from gaining access to full-text papers. However, fully or partially open-access journals allow all readers an opportunity to browse, read, and imagine how they might contribute to a similar format. In addition, through researching networking sites, such as ResearchGate (www.researchgate.net) which are free to use and set up a profile, authors often deposit their outputs in plain-text form (to avoid copyright infringement with the publishing journal) or can be contacted directly to request a copy of their article.

### Networking

Communication is the key to maintaining any well-oiled machine; and as such, networking with the operators of each of the three major cogs is how one accesses, and advances through, each stage of the process. The ‘active and passive networking’ in Fig. 1 alludes to the more formal, purposeful networking that one may initiate by approaching journals to offer their services as a peer reviewer, enrolling on workshops/courses concerned with editorial skills or through structured internship-like programmes. Passive networking occurs more serendipitously: discussions with a colleague about writing a paper together on a topic of shared interest, meeting someone in the coffee break who happens to be a peer reviewer/editor for a journal who then might invite you to join them on a future solo or group review. From small acorns grow mighty oaks, and it is possible that from these networking experiences the opportunities for assistant/deputy editor (and even more senior) roles are first sown! With this in mind, we offer insights about how one at an early stage of their academic career might engage with academic publication, and suggest that the order of authoring, reviewing, and editing is likely to be the most natural progression through the system for those who wish and gain entry to this academic field.

## Authoring

### First Steps: Get Writing!

Writing academically for publication can be a daunting process and might be considered reserved for those either with research to report, and/or those of a high status within the field. However, there are many different types of articles in academic journals—ranging from original research reports, to literature reviews, and commentaries. Personal view articles and practical guides—particularly in the field of medical education—are examples of accessible introductions to writing to early-career academics and can be undertaken as a single author or in collaboration with others. If for no other reason, these less intimidating article types offer the novice author an opportunity to acquire submission competency.

### Next Steps: Submitting Your Manuscript

Submitting a manuscript is usually done via an online submission system. Once a manuscript has been completed, it can be submitted via these online platforms according to the journal specifications. However, if there are any doubts as to the suitability of the paper, judicious contact of the journal editor can be made: requesting guidance and advice. While we would not encourage authors to flood the editor’s inbox unnecessarily, we have found the medical education community (including highly ranked editors) to be both friendly and approachable. In our experience, a carefully worded, professional email is unlikely to be met with malice. That said, writing and submitting manuscripts for publication can be a disarming experience. The acceptance rates for journals vary hugely; according to online information from two medical education journals, up to 80% of submissions are rejected [6, 7], and that include papers written by very experienced authors in the field. Improving your chances of success includes aligning your article to the journal, as described below.

For anyone considering writing an academic article, the style, format, and remit of the article (for a particular journal) are key information and are readily available on the journal website. Furthermore, many journals have mission statements or areas of interest—pitching this right is key to success. As a novice author, it is highly beneficial to lean on the support of others, particularly those who may be more experienced. Each author struggles with their own particular challenges in the writing process: for some it is prioritizing the time for writing, for others the act of putting words on paper can feel like a highly laborious task. Regardless, of the individual challenges faced, collaborative writing or informal peer review offers opportunities to improve your writing craft, while building a valuable academic network [8].

### Final Steps: The Small Print

Manuscripts may be associated with publication costs. These publication costs vary greatly, ranging from zero to thousands of dollars, depending on the journal and its particular publication model. This may be a particular challenge in the field of medical education, where researchers do not usually have access to large grants or research budgets, to assist with covering such fees. Authors from resource-constrained settings or institutions with pre-existing agreements may be eligible for fee discounts or waivers, and it is certainly a worthy effort to pursue this option, should it be available. Finally, having a better understanding of the peer-review process can be extremely helpful when considering your own writing; peeking behind the curtain and taking a closer look at how the editing and reviewing cogs work will no doubt reveal useful insights to smooth one’s own publication process.

## Reviewing

The peer-review system is an essential part of scholarship which serves to assess and improve manuscripts [2, 9]. This applies both to those manuscripts which are published and to those which are rejected for publication. Given that peer reviewers are selected and contacted by the editorial team to review a specific paper (usually one which aligns with their expertise and/or interest), early-career academics are unlikely be ‘called upon’ for this important task. However, from our experience as interns, we have been told frequently that peer reviewers are often in short supply. Whilst there are individuals who are keen and interested, time pressures often dictate that this particular task is not at the top of their priority list; and therefore, the invitation is often declined. Therefore, early-career academics are perhaps one of the best options to keep the peer-review pool afloat, but can be plagued by questions around what peer review is, how to perform a review, and whether their ‘opinion’ really counts? Existing literature [10] provide useful tips and guidance for those who are new to reviewing manuscripts. These papers are excellent guides as to *how to conduct* a review, but do not go into detail of how does one *become* a reviewer. Azer et al. briefly mention that a reviewer may invite other colleagues to collaborate on reviews, but none of the other entry points to reviewing is mentioned [10]. We expand on collaborative reviews below, and outline other mechanisms for scholars to access reviewing opportunities.

### Option 1: Traditional Peer Review

Volunteering your skills to be a reviewer [11] is an excellent way to start, particularly for those early-career researchers who may not yet have an extensive publication footprint. Most reputable journals have contact details available online, with some journals specifically inviting emails from interested potential reviewers. Indeed, this was the approach we took in our early careers. Therefore, the main challenge here is not necessarily *becoming* a peer reviewer (far from it—you will likely be welcomed by any journal to which you offer your services) but perhaps knowing how to gain experience and confidence in your own abilities as a peer reviewer of others’ work (with the additional pressure of doing so in a timely manner).

### Option 2: Group Peer Review

Traditionally, reviewing is a solitary academic activity. Group review, also termed collaborative or team-based review, runs in opposition to this. In this format, groups can be formed (either face-to-face at individual institutions or virtually); with the aim of collaborating on peer reviews. Some journals even encourage such practices and invite reviewers to disclose the names of colleagues who participated in group reviews, so that they may be appropriately acknowledged. The emerging literature suggests that collaborative reviews are an excellent opportunity for team-based learning, and tends to generate more robust and balanced reviews [12–14]. In particular, we propose that collaborative reviews may act as a means to allow junior or less-experienced colleagues an opportunity to navigate the review process within the safety of a community of practice. Furthermore, the process becomes more efficient for those who have workload pressures; by creating a specific time and space for group review, and with the additional resources within the group, reviews may be easily turned around before deadlines.

### Option 3: Open-Peer Review

As a more informal route into reviewing, one might consider engaging with open-peer review journals [15]. These utilise a post-publication review system, whereby the submitted manuscript undergoes an initial screening by the editor(s) before being published online. Experts in the field are invited to review the manuscript and submit their reviews directly online. This platform allows those less experienced with the review process to watch as it unfolds—comparing their own evaluation of the manuscript with that of the experts. Furthermore, comments can be left on the manuscript which also offer a forum for discussion around the paper which also may be of educational value.

## Editing

Editors are the gatekeepers of the journal; there are different roles and levels of responsibility attached to the different iterations (editor-in-chief, deputy editor, assistant editor, etc.) but all have an important role to play in overseeing the ‘editing’ and ‘reviewing’ cogs. Despite the title, editors are not often required to make changes or improvements to a submitted manuscript—they are editing the *journal* itself by curating its content. A common model for this can be considered as a network of deputy/assistant editors who are connected to the central editor-in-chief.

### First Step: Editorial Experience

Early-careers academics interested in getting hands-on experience within the editorial process may struggle to find an accessible route [16]. A handful of medical education journals run successful internship programmes to satisfy this need. They tend to run on an annual basis, and are an open application to potential candidates from all over the world with a diverse range of professional backgrounds. The number of interns appointed each year is very limited, and therefore many who apply would not be successful (at least the first time around). Both authors on this paper, HC and LG, applied for an editorial internship multiple times before being successfully selected. However, for those who are selected for editorial internship programmes, the opportunities to ask questions, discuss, and work alongside editors to better understand how a journal ‘functions’ are a high privilege.

### Next Steps: Associate Editor/Deputy Editor

Given the responsibility bestowed upon editors, they are often individuals who are at the peak of their career: experienced, well-published, and well-known in the community. The deputy/assistant editors are assigned submitted manuscripts that pass the initial screening performed by the Editor in Chief. They conduct the ‘reviewing’ process by inviting peer reviewers, collating their responses, and, in conjunction with the deputy/assistant editor’s own evaluation, return a recommendation to the editor-in-chief regarding whether to accept or reject the manuscript for publication.

### Final Step: Editor in Chief

The Editor in Chief has the last word on journal decisions. They are most often a renowned individual in their area of expertise with a wide range of editorial experience usually afforded by multiple previous associate/deputy editor positions for several different journals. From our intern experience, the authors acknowledge that the Editor in Chief relies on the insights from their deputy/assistant colleagues: individuals they trust in a professional capacity (and who also have excellent credentials). Whilst the final decision lies with the Editor in Chief, a successful and efficient publication process is likely to be underpinned by a collaborative editorial team effort.

## Conclusion

The academic publication system for medical education journals can seem like a members-only club which is difficult to access for newcomers to the field. Whilst the medical education community is generally encouraging of inclusivity, we believe that more can be done to invite new members, and hence the aim of this article.

There are opportunities for those at an early stage of their academic career to become involved, but these are not readily advertised. Whilst we are aware that there are factors that limit the opportunities for involvement in the publication process, particularly to people from non-Westernised countries, we firmly believe there are ways in which enthusiastic newcomers may dive in—and furthermore we encourage them to do so.

To safeguard the peer-review process for the future two things must occur; firstly, as championed in this article, new peer reviewers (and editors of the future) must be trained to navigate the process and to conduct rigorous, fair, and constructive reviews to uphold the high standards associated with the peer-review system, particularly with a renewed focus on ensuring equity, diversity, and inclusion across scholarly publications [17–19]. Secondly, existing and experienced researchers and educators must continue to oversee the process whilst encouraging, inviting, and inspiring the next generation of early-career educators and researchers to safeguard the future of this important repository of research dissemination and knowledge exchange.

We hope this article serves as an empowering, practical guide to breaking into the academic publication system, but moreover we are keen to ensure that academic publication has longevity; and therefore, the next generation must be nurtured. Therefore, this article should be considered a ‘call to action’ to the capable, motivated, and interested medical educationalists from across the world. As we enjoy the rise of medical education as its own discipline, scholarship must keep up with the pace. Your academic discipline (and its ability to disseminate good practice and evolve with the ever-changing healthcare landscape) needs you!
